# Unveiling the global nexus: Pandemic fear, government responses, and climate change-an empirical study

**DOI:** 10.1016/j.heliyon.2023.e23815

**Published:** 2023-12-29

**Authors:** Sabeeh Ullah, Sajid Rahman Khattak, Rezwan Ullah, Mohammad Fayaz, Heesup Han, Sunghoon Yoo, Antonio Ariza-Montes, António Raposo

**Affiliations:** a Institute of Business and Management Sciences, The University of Agriculture, Peshawar, Pakistan; b School of Management and Economics, Beijing Institute of Technology, Beijing 100081, China; c College of Hospitality and Tourism Management, Sejong University, Seoul 05006, South Korea; d Audit Team, Hanmoo Convention (Oakwood Premier), 49, Teheran-ro 87-gil, Gangnam-gu, Seoul 06164, South Korea; e Social Matters Research Group, Universidad Loyola Andalucía, C/Escritor Castilla Aguayo, 4, 14004 Córdoba, Spain; f CBIOS (Research Center for Biosciences and Health Technologies), Universidade Lusófona de Humanidades e Tecnologias, Campo Grande 376, 1749-024 Lisboa, Portugal

**Keywords:** Pandemic fear, Government responses, SARS-CoV-2, Climate change

## Abstract

This study examined the relationships between pandemic fear, government responses, and climate change using a time-series dataset from January 1, 2020, to December 31, 2020. By employing an auto-regressive distributed lag (ARDL) approach, the results revealed that pandemic fear significantly impacts climate change, while government responses to COVID-19 negatively influence climate change in the long run. Climate change and government responses significantly positively affect pandemic fear in the long run. Moreover, we found a bidirectional causality between government responses and climate change, unidirectional causality from government responses to pandemic fear, and no Granger causality between pandemic fear and climate change. Our findings have some important policy implications. Governments must encourage coordination, enhance crisis responses, and consider revising economic metrics to maintain environmental sustainability. The COVID-19 experience can inform strategies for reducing CO2 emissions and investing in green economies and healthcare to prepare for future challenges.

## 1 Introduction

In the last month of 2019, a new respiratory disease called severe acute respiratory syndrome Coronavirus-2 (SARS-CoV-2) (hereafter COVID-19) appeared in China, rapidly propagating to almost all parts of the world in a few months. As a result, millions of deaths and infected cases have been reported worldwide due to COVID-19, making it one of the leading causes of death [1]. On March 11, 2020, the World Health Organization (WHO) announced the COVID-19 outbreak as a global pandemic.

The increasing propagation of COVID-19 has built unbelievable and unprecedented situations [2] and created psychological fear, stress, and other health issues [3]. To reduce the increasing propagation of novel coronavirus, governments imposed stringent measures like isolation of symptomatic individuals, closure of schools and markets, bans on mass gatherings, and home confinements [4,5], reducing unnecessary patient burden on hospitals [6]. Also, various types of sports, culture, and religious events, industries operations were closed down, and all types of transportation were cancelled [4]. The governments took all these stringent measures to reduce the transmission of the virus, which adversely affected the global economy, everyday life, and work [4,7,8] and caused general disruptions in societies [3]. In addition, the pandemic response has become an issue of high interest [1].

On the contrary, the COVID-19 pandemic also positively and significantly affected the global environment. Due to the closure of industry operations, restrictions on air and road transportation, stoppage of all types of construction, and reduction in fossil fuels and other toxic tiny suspended particles, zero emissions of CO2 were achieved. Likewise, researchers documented that due to diminished vehicular traffic and industrial activity, CO2 emissions and other pollutants were reduced during the COVID-19 pandemic [5,[9], [10], [11]]. In addition, the International Energy Agency (IEA) (2020) reported that greenhouse gas (GHG) emissions could fall by 8 % globally. Moreover [12], argued that the air quality had improved during the pandemic in India. However, during early February and mid-March 2020, CO2 emissions were reduced by about 18 % (250 m tonnes) in China and 390 m tonnes in Europe [4].

Understanding the empirical nexus between the pandemic fear associated with confirmed deaths and cases due to COVID-19, government responses, and climate change becomes paramount as the world copes with these complex issues. Do heightened levels of pandemic fear lead to environmental policies? How do governments' responses influence climate change adaptation and mitigation efforts? This study aims to address these questions and provide a deeper understanding of the empirical nexus between pandemic fear, government responses, and climate change.

The study makes several distinctive contributions to the contemporary literature. First, various environmental and socio-economic changes and health issues were seen worldwide during this pandemic [1,12]. Despite the deaths worldwide due to the disease, each country imposed a smart or complete lockdown to reduce the coronavirus's exponential rise, leading to psychological fear [13]. However, on the positive side, the nation's lockdowns due to the pandemic also brought some positive environmental changes worldwide. Regarding causes and consequences, climate change and the COVID-19 pandemic share striking similarities [14,15]. Keeping both aspects of the unprecedented COVID-19 pandemic, government responses, and CO2 emissions reduction necessitates advancing our understanding of how pandemic fear and government responses influence climate change-related policies and practices.

Second, contemporary researchers reported that the pandemic offers a rapid learning experience to prevent global climate change [7,[14], [15], [16], [17], [18], [19]]. In addition [20], reported that the nexus of the pandemic and climate change has diverted attention to CO2 emission reductions and public health responses. Therefore, understanding the empirical link between the Coronavirus pandemic and climate change is essential for companies, consumers, and societies at large [21], helping to maintain public support for climate policies [14].

Finally, most prior studies are exploratory, e.g. Refs. [4,7,14,19,22,23]. Therefore, more research is needed to investigate CO2 emissions during the Coronavirus pandemic [22] and government responses that lead to negative and positive effects. Hence, the study empirically examines the causal relationship between global fear due to the COVID-19 pandemic, government responses to the virus, and climate change. This is the study's novelty; as to our understanding and the extant literature, none of the studies before examined this nexus.

## 2 Literature and hypothesis development

### 2.1 Theoretical support

Integrating Behavioral and Complex Systems Theory offers a holistic framework for understanding the empirical nexus of pandemic fear, government responses, and climate change. The two conflicting behavioral theories of the “finite pool of worry” and “affect generalization” hypotheses provide valuable insights into understanding how people and states allocate their attention and concerns to different issues and threats. According to Weber's finite pool of worry, people have limited resources for worrying and, as a result, avoid dealing with multiple problems simultaneously. Increased fear about one factor reduces concerns about others [24,25]. People worry more about the COVID-19 pandemic and neglect climate change issues [26]. [27] documented that during the COVID-19 pandemic, framing climate change as a secondary issue reduces support for climate change mitigation. The COVID-19 pandemic delayed climate policy implementation and changed priorities from climate change actions to COVID-19 pandemic responses [28]. Consistent with this theory, the emergence of the COVID-19 pandemic may temporarily dominate the finite pool of worry, leading to heightened concerns about public health and safety, which may overshadow concerns about climate change during the crisis.

On the contrary, the “affect generalization” hypothesis states that a rise in fear about a threat may increase concern about the other issue [29,30]. By framing pandemic fear and government responses in ways that evoke positive attitudes and emotions, such as unity, responsibility, and resilience, and generalizing these positive emotions to issues like climate change and building support for related policies [31]. reported that concern about the COVID-19 pandemic enhances climate change concern, supporting the “affect generalization” hypothesis. The hypothesis can also be reinforced if people are motivated to obey government responses/actions during the pandemic, which might generalize to other activities that promote environmental sustainability [32]. documented that the COVID-19 pandemic reduces CO2 emissions due to government responses (the shutdown of the aviation sector and local transport) by improving air quality for the next generation [33]. This hypothesis supports examining how responses in one domain, such as pandemic fear and government responses, can be generalized to other domains/actions that enhance environmental sustainability.

The complex system theory emphasizes the interconnectedness of various components within a system. The complex system theory recognizes that changes in one part can have ripple effects throughout the entire system. Complex system theory stresses the dynamic nature of causality and intricate and emergent behaviours in a system that can be used in a changing environment [34]. These interactions provide rise to new phenomena (for instance, pandemic fear, government response, and climate change) at the system level. The COVID-19 crisis analysis mobilized the concepts involved in complex systems analysis [35]. This theory provides a critical lens to understand how pandemic fear, government responses, and climate change are interrelated and influence one another.

These theoretical foundations allow us to empirically examine the dynamic, interdependent, and often unpredictable relationships among these complex global phenomena of pandemic fear, government responses, and climate change, offering insights into how changes in one component can affect the entire system and its behavior.

### 2.2 Hypothesis development

#### 2.2.1 Pandemic fear and climate change

The severity and fast-growing nature of the coronavirus influenced every aspect of human life, both socially and economically. The increasing mortality and infection rates created fear of losing control, invisibility, and insecurity. Fear is a fundamental personal and social feeling and a central element during emergencies and crises [36]. Despite the adverse effect of the COVID-19 pandemic on the economy and health systems, the world has noticed some positive influence of the COVID-19 pandemic in the form of environmental recovery [37]. Around the globe, cleaner waterways, air, and skies are evident in some regions [38]. [39] documented that for the first time in decades, Indians see the Himalayas. In the first wave of the COVID-19 pandemic, nations imposed various lockdown strategies to overcome the rapid spread of coronavirus. As a result, the global economy slowed due to a decline in major manufacturing and industrial activities, energy demands, ground and air transportation, and outside movements of people from home [40]. These declines prompted a sharp reduction in greenhouse gas emissions (i.e., CO2) [41]. [40] reported a reduction in CO2 emissions with the increase in COVID-19 confirmed cases. Similarly [42], documented a 5.8 % reduction in CO2 emissions globally during the first wave of the COVID-19 pandemic. In addition [43], found an adverse relationship between the COVID-19 pandemic and CO2 emissions. Based on these arguments, the study hypothesized the following:

H1 COVID-19 pandemic fear *negatively affects CO2 emission.*

#### 2.2.2 Pandemic fear and government responses

The rapid propagation of coronavirus across nations and high pressure on the health system create fear of the coronavirus both among people and governments. Fear of the pandemic is the main factor of protective behavior compliance during the pandemic [[44], [45], [46]]. The pandemic fear forced most governments to induce various non-pharmaceutical steps to overcome the spread of the virus. These non-pharmaceutical steps were related to social distancing, closure of workplaces and schools, meeting restrictions, public transport, and national and international travel [47]. In September 2020, approximately 186 countries imposed movement restrictions, of which 82 countries induced regional and national lockdowns [48]. In addition [49], argued that a wide range of responses from the government increased with the spread of the coronavirus. Likewise [50], documented that with the rise of deaths and infection rate of COVID-19, government responses to curtail it also increased. The COVID-19 pandemic necessitates government responses to be sharply enacted to stop the propagation of the coronavirus [51]. Based on this, the study hypothesizes.

H2 *The increase in the* COVID-19 *pandemic necessitates government responses.*

#### 2.2.3 Government responses and climate change

To control the propagation of coronavirus, various governments implanted severe containment strategies. The government responded with minimal human activities, reducing CO2 emissions in different parts of the world [41]. Temporary lockdowns across the globe led to the abrupt stoppage of main transportation and economic activities, prompting a quick decline in CO2 emissions [40]. Moreover, people have witnessed positive changes in the local environment due to these reduced movements and global production. Thus, the study hypothesizes as follows:

H3 *Government responses to* the COVID-19 *pandemic positively affected climate change.*

## 3 Materials and methods

### 3.1 Data and variables

The study employed multiple time series data sources from January 1, 2020 to December 31, 2020. First, for the Global Pandemic Fear Index, daily data on COVID-19 cases and deaths in each country were obtained from the “Our World in Data” and the Center for Systems Science and Engineering (CSSE) at Johns Hopkins University. Second, for government responses to the pandemic, the Oxford COVID-19 government response tracker (OxCGRT) index was used to collect information regarding governments' responses to the pandemic [52]. Lastly, following [53], CO2 emission is a proxy for climate change. Due to the unavailability of real-time daily global CO2 emissions data, we used the published estimated daily data of global CO2 emissions, made public by Ref. [5] from January 1, 2020 to December 31, 2020. Table 1 details the descriptions of the variables, the proxy used, and the data sources.

**Table 1:** Variables definition, proxies used and data sources.

Variables	Proxy	Definition	Used by	Data source
Pandemic Fear	Confirmed Cases	Number of confirmed new COVID-19 infected cases	[26,31]	Our World in Data https://ourworldindata.org/coronavirus and CSSE at Jhon Hopkin University https://systems.jhu.edu/tracking-covid-19/
	Confirm Deaths	Number of confirmed new COVID-19 deaths		
Government Responses	Governments Response Index	Government responses to COVID-19 are measured by nine indicators, including closure of schools, workplaces, public transport, cancelling public events, home confinement orders, international travel controls, restrictions on domestic movement and gathering size, and public information campaigns.	[31,49]	Oxford COVID-19 government response tacker (OxCGRT). https://github.com/OxCGRT/covid-policy-dataset
Climate Change	CO2 emissions	Mean emissions in MtCO2/day	[5]	Global Carbon Project https://www.icos-cp. eu/global-carbon-budget-2019

### 3.2 Operationalization of variables

#### 3.2.1 Construction of the global fear index (GFI)

Following [54], a composite global fear index (GFI) was constructed on two factors on a scale of 0–100, which is as follows:

##### 3.2.1.1 Confirmed cases index (CCI)

Contemporary research documented that the spread of coronavirus is mainly due to respiratory droplets and human-to-human transmission [22,55]. In contrast, climate conditions are the main predictor of Coronavirus disease transmission [56]. Therefore, an increasing number of confirmed cases create global fear that leads to stringent measures of lockdown by governments, which impact the environment positively. We constructed a CCI as follows:

(1) $${CCI}_{t}=\frac{\sum_{i=1}^{N}C_{it}}{\sum_{i=1}^{N}\left(C_{it}+C_{i\left(t-14\right)}\right)}\times 100$$

Where ${CCI}_{t}$ is the Coronavirus cases index at time t, $C_{it}$ is the COVID-19 confirmed cases for country i at time t, and $C_{i\left(t-14\right)}$ is the confirmed COVID-19 cases for country i at the start of the incubation period t-14.

##### 3.2.1.2 Confirmed deaths index (CDI)

Prior research documented that more exposure to polluted air increases the vulnerability to COVID-19, increasing the death rate from the disease [57]. The excessive deaths associated with the COVID-19 pandemic increased global fear and inducted lockdowns, which reduced CO2 emissions and improved environmental quality. Similar to CCI, we computed CDI as follows:

(2) $${CDI}_{t}=\frac{\sum_{i=1}^{N}D_{it}}{\sum_{i=1}^{N}\left(D_{it}+D_{i\left(t-14\right)}\right)}\times 100$$

Where ${CDI}_{t}$ is the confirmed deaths index at time t, $D_{it}$ is the COVID-19 confirmed deaths for country i at time t, and $D_{i\left(t-14\right)}$ is the COVID-19 confirmed deaths for country i at the start of incubation period t-14.

##### 3.2.1.3 Composite global fear index (GFI)

To obtain a composite global fear index (GFI), we assigned equal weights to each index in Eqs. (1), (2) and computed the GFI as follows:

(3) $${GFI}_{t}=\frac{1}{2}\left({CCI}_{t}+{CDI}_{t}\right)$$

#### 3.2.2 Government responses

For the government responses to the COVID-19 pandemic, simple averages of each country's response index value for a corresponding day were calculated as follows:

(4) $${GRI}_{t}=\frac{1}{N}\sum_{i}^{N}I_{i}$$

Where ${GRI}_{t}$ is the government response index at time t and $I_{i}$ is each country's response index value.

#### 3.2.3 Global climate change

For climate change, we used CO2 emissions as a proxy because they are the primary driver of climate change and are of critical importance for understanding global climate change [5]. Ref. [5] calculated global as well as country-level CO2 emissions on a confinement index basis using the following Equation (5):

(5) $$\Delta {CO}_{2}={CO}_{2}\times \delta S\times \Delta A$$

**Fig. 1:**
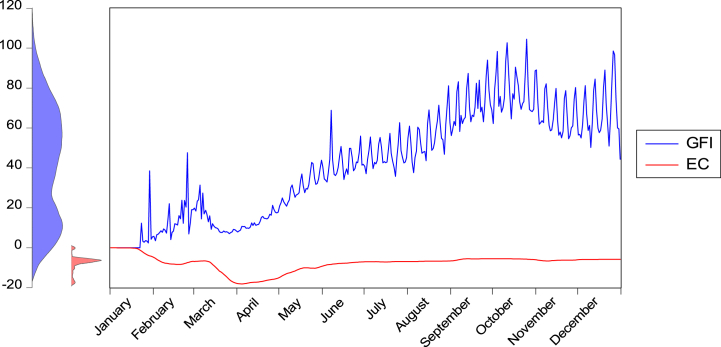
Global pandemic fear and CO2 emission during the COVID-19 outbreak.

**Fig. 2:**
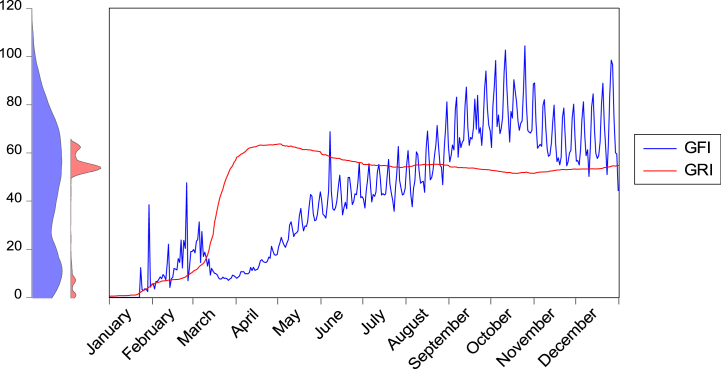
Global Pandemic Fear and government responses during the COVID-19 outbreak.

**Fig. 3:**
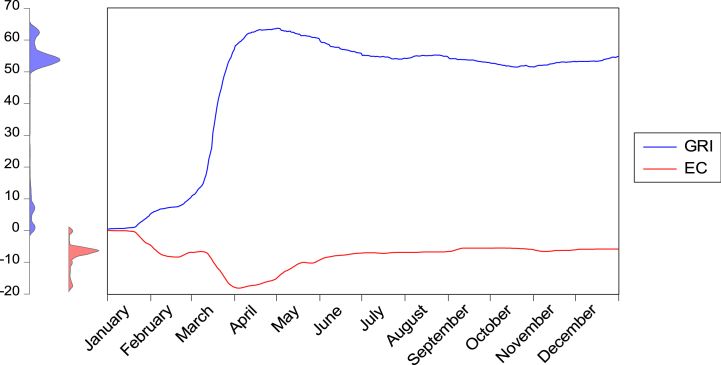
Government responses and CO2 emission during the COVID-19 outbreak.

Where $\Delta {CO}_{2}$ is the daily emissions change, ${CO}_{2}$ is the mean daily emission, δS is the fractional change of each sector's emissions, and ΔA is the fractional change in the activity level compared with the pre-COVID-19 pandemic. Fig. 1, Fig. 2, Fig. 3 outline the graphical relationship among the variables during the COVID-19 outbreak.

### 3.3 Econometric methods and procedure

To estimate the empirical nexus among global pandemic fears, government responses, and climate change, we employed a series of econometric tests on Eq. (6):

(6) $${GE}_{t}=\alpha +\beta_{1}{GFI}_{t}+\beta_{2}{GRI}_{t}+\varepsilon_{t}$$

Where ${GE}_{t}$ is the estimated global CO2 emissions, ${GFI}_{t}$ is the global fear index calculated in Eq. (3), ${GRI}_{t}$ is the government responses index measured in Eq. (4), and $\varepsilon_{t}$ is the error term.

#### 3.3.1 Unit root test

For the time series framework, we ascertain the order of integration for the variables before checking the causality test. For this purpose, different tests were employed, namely Augmented Dickey-Fuller (ADF) [58,59], Dickey-Fuller Generalized Least Square (DF-GLS) [60], and Phillips Perron (PP) tests [61]. Null hypotheses of these tests were tested using the following regressions Equations (7), (8), (9):

(7) $$\text{ADF}\;\text{test}:\Delta z_{t}=\Psi z_{t-1}+\sum_{j=1}^{p}\eta_{j}\Delta z_{t-1}+\mu_{t}$$

(8) $$\text{ADF}‐\text{GLS}\text{test}:\Delta z_{t}^{d}=\Psi z_{t-1}^{d}+\sum_{j=1}^{p}\eta_{j}\Delta z_{t-j}^{d}+\mu_{t}$$

(9) $$\text{PP}\;\text{test}:\Delta z_{t}=\Psi z_{t-1}++\mu_{t}$$

where z is the variable, Δ is the difference operator, d is de-trended, $\mu_{t}$ is the disturbance term. For $\Psi =\varphi -1,H_{0}:\varphi =1\;and\;H_{1}:\varphi <0$.

#### 3.3.2 ARDL bound test

To check the long-run association among the study variables $\left\lfloor {CE}_{t},{GFI}_{t\;},{GRI}_{t\;}\right\rfloor$, we employed the ARDL bound test procedure developed by Refs. [62,63], which is expressed as follows:

(10) $$\left[\begin{array}{c} \Delta {CE}_{t}\\ \Delta {GFI}_{t}\\ \Delta {GRI}_{t} \end{array}\right]=\left[\begin{array}{c} \alpha_{01}\\ \alpha_{02}\\ \alpha_{03} \end{array}\right]+\left[\begin{array}{ccc} \beta_{11} & \beta_{12} & \beta_{13}\\ \beta_{21} & \beta_{22} & \beta_{23}\\ \beta_{31} & \beta_{32} & \beta_{33} \end{array}\right]\left[\begin{array}{c} {CE}_{t-1}\\ {GFI}_{t-1}\\ {GRI}_{t-1} \end{array}\right]+\sum_{i=1}^{p}\left[\begin{array}{ccc} \delta_{1i} & \varphi_{1i} & \eta_{1i}\\ \delta_{2i} & \varphi_{2i} & \eta_{2i}\\ \delta_{3i} & \varphi_{3i} & \eta_{3i} \end{array}\right]\left[\begin{array}{c} \Delta {CE}_{t-i}\\ \Delta {GFI}_{t-i}\\ \Delta {GRI}_{t-i} \end{array}\right]+\left[\begin{array}{c} \mu_{1t}\\ \mu_{2t}\\ \mu_{3t} \end{array}\right]$$

The ARDL bound test used modified t-statistics and joint F-statistics for determining the long-run association and normalization of variables. Eq. (10) is a vector equation of three equations denoted as $F_{CE}\left(CE|GFI,GRI\right),F_{GFI}\left(GFI|CE,GRI\right),{and\;F}_{GRI}\left(GRI|CE,GFI\right)$. Here, for no co-integration, the null hypothesis is $H_{0}:\beta_{i1}=\beta_{i2}=\beta_{i3}=0$ against the alternate hypothesis $H_{1}:\beta_{i1}\neq \beta_{i2}\neq \beta_{i3}\neq 0$, where i = 1, 2, 3. Using the Schwarz information criterion (SIC), we used a maximum of 4 lags for the ARDL vector error correction model (VECM).

#### 3.3.3 Long-run relationship

The presence of co-integrating relationships in two equations indicates that a long-run relation can be estimated as follows:

(11) $${CE}_{t}=\alpha_{01}+\sum_{i=1}^{p}\beta_{1i}{CE}_{t-i}+\sum_{i=0}^{q}\beta_{2i}{GFI}_{t-i}+\sum_{i=0}^{r}\beta_{3i}{GRI}_{t-i}+\mu_{t}$$

(12) $${GFI}_{t}=\alpha_{01}+\sum_{i=1}^{p}\beta_{1i}{GFI}_{t-i}+\sum_{i=0}^{q}\beta_{2i}{CE}_{t-i}+\sum_{i=0}^{r}\beta_{3i}{GRI}_{t-i}+\mu_{t}$$

Eq. (11) is estimated for ARDL (3, 0, 1), and Eq. (12) is calculated using ARDL (4, 0, 0). The lag selections in these equations are based on the SIC criteria.

#### 3.3.4 Granger causality

Following [[64], [65], [66], [67]], the short-run dynamic parameters excluding error correction were estimated for Eq. (15), where GRI is a predictor, and found no co-integration among the variables. However, Eq. (13) and Eq. (14), in which CE and GFI are dependent variables respectively, are co-integrated and were estimated by including an error correction term through the following equations:

(13) $${\Delta CE}_{t}=\alpha_{01}+\sum_{i=1}^{p}\beta_{1i}{\Delta CE}_{t-i}+\sum_{i=0}^{q}\beta_{2i}{\Delta GFI}_{t-i}+\sum_{i=0}^{r}\beta_{3i}{\Delta GRI}_{t-i}+\gamma {ECT}_{t-1}+\mu_{t}$$

(14) $${\Delta GFI}_{t}=\alpha_{01}+\sum_{i=1}^{p}\beta_{1i}{\Delta GFI}_{t-i}+\sum_{i=0}^{q}\beta_{2i}{\Delta CE}_{t-i}+\sum_{i=0}^{r}\beta_{3i}{\Delta GRI}_{t-i}+\gamma {ECT}_{t-1}+\mu_{t}$$

(15) $${\Delta GRI}_{t}=\alpha_{01}+\sum_{i=1}^{p}\beta_{1i}{\Delta GRI}_{t-i}+\sum_{i=0}^{q}\beta_{2i}{\Delta CE}_{t-i}+\sum_{i=0}^{r}\beta_{3i}{\Delta GFI}_{t-i}+\mu_{t}$$

Here ${ECT}_{t-1}$ is the error correction term, Δ is the difference operator, γ is the adjustment speed. Table 4 shows the findings of Eq. (13), (14), (15)), which are separately estimated using ordinary least squares regression.

## 4 Results and discussions

Table 2 illustrates the results of unit root tests, which indicate the non-stationary of all the variables at the level. At first, the results of these three tests are stationary and show that all the variables are integrated of order 1 (i.e., I (1)).

**Table 2:** Unit root tests.

Variables	ADF test		ADF-GLS test		PP test		I(d)
	Level	1st difference	Level	1st difference	Level	1st difference	
CE	−1.112	−3.632	−1.274	−3.634	−0.533	−3.212	I (1)
GFI	−1.185	−17.168	−1.355	−17.070	−0.809	−30.745	I (1)
GRI	−0.038	−3.416	−0.189	−3.408	0.469	−5.371	I (1)
Significance Level	Test Critical Values						
1 % level	−2.571		−2.571		−2.571		
5 % level	−1.942		−1.942		−1.942		
10 % level	−1.616		−1.616		−1.616		

The t-statistic and joint F-statistic, along with the critical values at 10 % and 5 % for the three equations, are reported in Table 3. Our results authenticate the existence of a long relationship when CE and GFI are used as dependent variables. The F-statistic and t-statistic for these two equations are higher than the critical values at I (1) bounded at 10 % and 5 %. Thus rejecting the null hypothesis (i.e. $H_{0}:\beta_{i1}=\beta_{i2}=\beta_{i3}=0$). However, there is no co-integration when GRI is used as a dependent variable.

**Table 3:** ARDL Bound test for co-integration.

Model	F-statistic		t-statistic		Decision	
$F_{CE}\left(CE|GFI,GRI\right)$	10.898		−4.628		Co-integration	
$F_{GFI}\left(GFI|CE,GRI\right)$	5.027		−3.868		Co-integration	
$F_{GRI}\left(GRI|CE,GFI\right)$	2.197		−2.351		No Co-integration	
Critical Values						
Significance Level	I (0)	I (1)	I (0)	I (1)		
10 %	3.17	4.14	−2.57	−3.21		
5 %	3.79	4.85	−2.86	−3.53		

Table 4 illustrates the outcomes of the long-run estimation coefficients of Eq. (11) and Eq. (12). In Model 11, the coefficient of GFI is significant and positively associated with CE at a 5 % level. In contrast, GRI is negatively related to CE at a 1 % level. The positive relation of GFI with CE supports the notion that more exposure to polluted air increases vulnerability to COVID-19, increasing the death rate from the disease [57]. In the long run, our result is also supported by Ref. [68], who argued that in response to the COVID-19 pandemic, CO2 emissions may increase in both the medium and long term. This rise could be attributable to the resumption of industrial and transportation activities after the lockdown [12,69]. Moreover, the coefficient of GRI has a significantly negative effect on CE, indicating that government responses for reducing COVID-19 transmission reduce CO2 emissions. Our results align with the prior studies [[3], [4], [5],^57^,^70^].

**Table 4:** Estimated coefficients from long-run ARDL Model.

Variables	Model 11	Model 12
	ARDL (3, 0, 1)	ARDL (4, 0, 0)
Const.	−0.029 (−4.140)***	3.442 (2.391)**
CE	–	0.587 (2.901)***
GFI	0.0003 (2.433)**	–
GRI	−0.032 (−5.694)***	0.142 (3.200)***
R-squared	0.993	0.914
Prob (F-statistic)	0.000	0.000
Durbin-Watson stat	2.101	2.156

Note: *** and ** denotes 1 % and 5 % significance level, respectively. Values in (.) indicate t-statistics. Here, we do not report the lag values of the dependent and exogenous variables.

Table 4 also reported a long-run result of Eq. (12) in which GFI is used as a dependent variable. Here, both the variables CE and GRI have a significant, positive influence on GFI. The positive effect of CE on GFI again supports the notion that more exposure to polluted air increases the vulnerability to COVID-19, increasing the death rate from the disease [57]. Similarly, the significantly positive coefficient of GRI with GFI indicates that increased government responses for reducing COVID-19 transmissions increase global fear. The results align with prior studies' outcomes [[71], [72], [73]], which documented that lockdowns due to COVID-19 may increase psychological fear of the disease.

In conclusion, the findings contribute to the contemporary literature by confirming the negative association of government responses to the COVID-19 pandemic with CO2 emissions, supporting environmental sustainability. The COVID-19 pandemic could be viewed as a rapid learning experiment to cope with climate change [14]. Similarly, a rise in CO2 emission enhances government responses. The results underscore the importance of government responses to the COVID-19 pandemic and link it to climate change risks and environmental sustainability. Moreover, increasing government responses to COVID-19 transmission reduction enhances psychological fear of the disease; these findings emphasize initiatives and policies to overcome behavioral tendencies and effectively respond to future pandemics and climate changes.

In Table 5, from Model 13, the long-run causal link of GFI and GRI with CE is also confirmed from the coefficient of ${ECT}_{t-1}$. The coefficient of ${ECT}_{t-1}$ is significantly negative at the 1 % level, implying that the long-run causality interactively flows through the error correction term from GFI and GRI to CE. The value of ${ECT}_{t-1}$ is −0.992, indicating that the adjustment speed to the equilibrium is very high after the shock. It means that a deviation of CO2 emissions from the level of equilibrium in the COVID-19 pandemic period will be adjusted by 99.2 % in the coming period. Our results supported the view of [74] that the reduction in CO2 emission during the COVID-19 pandemic was temporary; however, CO2 emission can be reduced in the long run by implementing appropriate regulations/actions at different levels around the globe. Likewise, in Model 14, the statistically significant coefficient of ${ECT}_{t-1}$ confirms the long-run casual flows from CE and GRI to GFI with an adjustment speed of 73.6 %. In Model 15, only CE is negative and significant at a 1 % level for the short run.

**Table 5:** Estimated coefficients from Eq. (13), (14), (15)).

	Model 13		Model 14		Model 15
Variables	SIC Lags (3, 0, 1)	Variables	SIC Lags (4, 0, 0)	Variables	SIC Lags (4, 0, 0)
C	0.001 (0.256)	C	0.210 (0.498)	C	0.017 (1.171)
D (CE (-1))	2.097 (11.844)***	D (GFI(-1))	0.455 (1.855)*	D (GRI (-1))	0.355 (6.610)***
D (CE (-2))	−1.262 (−5.621)***	D (GFI(-2))	−0.131 (−2.335)**	D (GRI (-2))	0.256 (4.676)***
D (CE (-3))	0.144 (1.966)**	D (GFI(-3))	−0.280 (−4.578)***	D (GRI (-3))	0.263 (4.781)***
D (GFI)	0.001 (2.214)**	D (GFI(-4))	−0.110 (−1.131)	D (GRI (-4))	−0.011 (−0.212)
D (GRI)	−0.021 (−2.812)***	D (CE)	2.553 (0.862)	D (CE)	−0.268 (−2.509)***
D (GRI (-1))	0.015 (1.706)*	D (GRI)	0.239 (0.236)	D (GFI)	0.002 (1.382)
ECT (-1)	−0.992 (−5.360)***	ECT (-1)	−0.736 (−2.945)***	–	–
R-squared	0.956		0.254		0.783
Adjusted R-squared	0.955		0.240		0.780
F-statistic	1105.882		17.207		213.211
Prob (F-statistic)	0.000		0.000		0.000
Durbin-Watson Statistic	1.992		2.292		1.983

Note: *,**, and ***shows 10 %, 5 %, and 1 % significance, respectively. Values in (.) denote t-statistics.

Table 6 reports the outcomes of pair-wise short-run Granger causality. In the short run, F-statistics values illustrate a bi-directional Granger causality between government responses to COVID-19 and CO2 emissions. These results support the view of prior studies that due to lockdown restrictions by each country in the first wave of coronavirus, global CO2 emissions were reduced [3,5,57,75]. Unidirectional Granger causality runs from government responses due to COVID-19 to global fear of the disease. Also, there is no Granger causality between global fear of pandemic and CO2 emission.

**Table 6:** Pair-wise granger causality.

Dependent variable	F-statistics			ECT t-statistics	Direction of causality
	CE	GFI	GRI		
CE	–	1.603	15.799***	−0.992***	GRI→CE
GFI	0.787	–	3.424***	−0.736***	GRI→GFI
GRI	12.076***	1.768	–	–	CE→GRI

Note: *** denotes significance at 1 %.

## 5 Conclusion

The study examined the empirical nexus among Pandemic fear, government responses, and climate change. For this purpose, we used world time-series data from January 1, 2020 to December 31, 2020. Using an ARDL approach, our results demonstrate that Pandemic fear significantly influences CO2 emission. In contrast, government responses to COVID-19 negatively affect CO2 emissions in the long-run equilibrium. The long-run causal relationship between pandemic fear and government responses to CO2 emission is also confirmed by the coefficient of ${ECT}_{t-1}$ with an adjustment speed of 99.2 %. Moreover, CO2 emission and government responses to COVID-19 significantly positively affect pandemic fear in the long run, with an adjustment speed of 73.6 %. Granger causality results confirm the existence of a bi-directional causality between government responses and CO2 emission and a unidirectional causality between government responses and pandemic fear. However, there is no Granger causality between pandemic fear and CO2 emission.

## 6 Policy implications

Our results have some important implications for government and policy-making bodies. First, the Coronavirus pandemic is a global health crisis that has shaken each country's economy and health system. The increased government responses to COVID-19 transmission reduction enhance psychological fear of the disease. Therefore, governments and policymakers should emphasize initiatives and policies to overcome behavioral tendencies and effectively respond to future pandemics and climate changes. Second, the interconnections between the COVID-19 pandemic, governmental responses, and climate change necessitate that system-level reflection and coordination are needed. Therefore, governments should revise the current metrics of gross domestic product with the green gross domestic product and economic well-being. Thirdly, The COVID-19 pandemic could be viewed as a rapid learning experiment to cope with climate change. Therefore, considering the importance of government responses to the COVID-19 pandemic, policymakers should formulate and implement measures to combat CO2 emissions and maintain environmental sustainability. Finally, governments must improve the health system and invest in the green economy to respond to future pandemics and ecological changes.

## 7 Limitations and future directions

Our study has some limitations that need to be covered in future studies. This study is limited to global-level data on the pandemic, government responses, and CO2 emission, which ignores the people's individual level behavior in the COVID-19 period and government responses; future studies could examine national and sectoral level COVID-19 pandemic, government response, and CO2 emission. Moreover, future studies may analyze the same variables in the panel data framework.

## Data availability

The data that support the findings of this study will be made available on request.

## Funding statement

This research received no external funding.

## CRediT authorship contribution statement

**Sabeeh Ullah:** Writing – review & editing, Writing – original draft, Visualization, Validation, Supervision, Software, Resources, Project administration, Methodology, Investigation, Funding acquisition, Formal analysis, Data curation, Conceptualization. **Sajid Rahman Khattak:** Writing – review & editing, Writing – original draft, Visualization, Validation, Supervision, Software, Resources, Project administration, Methodology, Investigation, Funding acquisition, Formal analysis, Data curation, Conceptualization. **Rezwan Ullah:** Writing – review & editing, Writing – original draft, Visualization, Validation, Supervision, Software, Resources, Project administration, Methodology, Investigation, Funding acquisition, Formal analysis, Data curation, Conceptualization. **Mohammad Fayaz:** Writing – review & editing, Writing – original draft, Visualization, Validation, Supervision, Software, Resources, Project administration, Methodology, Investigation, Funding acquisition, Formal analysis, Data curation, Conceptualization. **Heesup Han:** Writing – review & editing, Writing – original draft, Visualization, Validation, Supervision, Software, Resources, Project administration, Methodology, Investigation, Funding acquisition, Formal analysis, Data curation, Conceptualization. **Sunghoon Yoo:** Writing – review & editing, Writing – original draft, Visualization, Validation, Supervision, Software, Resources, Project administration, Methodology, Investigation, Funding acquisition, Formal analysis, Data curation, Conceptualization. **Antonio Ariza-Montes:** Writing – review & editing, Writing – original draft, Visualization, Validation, Supervision, Software, Resources, Project administration, Methodology, Investigation, Funding acquisition, Formal analysis, Data curation, Conceptualization. **António Raposo:** Writing – review & editing, Writing – original draft, Visualization, Validation, Supervision, Software, Resources, Project administration, Methodology, Investigation, Funding acquisition, Formal analysis, Data curation, Conceptualization.

## Declaration of competing interest

The authors declare that they have no known competing financial interests or personal relationships that could have appeared to influence the work reported in this paper.
